# APP-C31 pathology as a target in neurodegenerative diseases

**DOI:** 10.1186/s12929-026-01216-3

**Published:** 2026-02-04

**Authors:** King Chi Yip, Woon Fei Ho, Yang Liu, Gavin Stewart Dawe

**Affiliations:** 1 Department of Biomedical Science, College of Biomedicine, City University of Hong Kong, Kowloon, 999077 Hong Kong China; 2 Department of Pharmacology, School of Medicine, Nanjing University of Chinese Medicine, Nanjing, 210023 Jiangsu China; 3 Department of Pharmacology, Yong Loo Lin School of Medicine, National University of Singapore, Singapore, 119077 Singapore; 4 Neurobiology Programme, Life Sciences Institute, National University of Singapore, Singapore, 119077 Singapore; 5 Healthy Longevity Translational Research Programme, Yong Loo Lin School of Medicine, National University of Singapore, Singapore, 119077 Singapore; 6 Precision Medicine Translational Research Programme, Yong Loo Lin School of Medicine, National University of Singapore, Singapore, 119077 Singapore

**Keywords:** Amyloid precursor protein (APP), APP-C31 fragments, Neurodegenerative diseases, Therapeutic target

## Abstract

Neurodegenerative diseases (Alzheimer’s disease, Parkinson's disease, Huntington’s disease, etc.) are caused by the progressive loss of neurons, which affects many people worldwide. Therefore, many efforts have focused on neurodegenerative disease mechanisms and therapeutic strategies. Moreover, amyloid precursor proteins and their cleaving products, including APP-C31, may play important roles in neurodegeneration. This review provides a comprehensive introduction to the structure, neurotoxicity, regulatory mechanism, and relevance of APP-C31 to clinical diseases and its therapeutic potential as a drug target. This work will bridge the gap in our understanding of the function of APP-C31, which provides an experimental basis for neurodegenerative disease therapeutics. Meanwhile, a hypothesis is postulated that the APP-C31 functions not merely as a byproduct of caspase cleavage, but as the critical "central executioner" bridging upstream triggers and downstream neurodegeneration. Diverse upstream stressors, initiate the cascade to generate APP-C31. Once generated, C31 acts as a multi-functional signalling hub driving four distinct pathogenic pathways. Consequently, APP-C31 is hypothesized to be the essential mediator that amplifies these molecular damages into macroscopic failures.

## Introduction

Neurodegenerative diseases affect millions of individuals globally, particularly within the aging population. Alzheimer’s disease (AD) and Parkinson’s disease (PD) are among the most prevalent. These conditions are marked by the progressive degeneration and dysfunction of the central nervous system (CNS). Recent studies suggest that the amyloid precursor protein (APP) plays a crucial role in the pathology of various neurodegenerative disorders [1, 2].

APP is a large, membrane-spanning glycoprotein [3] and a member of the conserved type 1 membrane protein family [4]. It undergoes complex proteolytic processing to yield various fragments, some of which are neurotoxic [5]. Notably, amyloid β (Aβ) [6] and the 31-amino-acid C-terminal fragment known as APP-C31 [7] have been implicated in neurodegenerative processes. While Aβ has been the focus of many studies and reviews due to its role in amyloid plaque formation, APP-C31 has received comparatively less attention despite mounting evidence of its neurotoxicity.

Interestingly, APP also has important physiological roles in the nervous system, such as facilitating neurite outgrowth, synaptogenesis, and cell adhesion. Secreted forms like sAPPα have been shown to promote cell proliferation and exert neuroprotective effects [3, 4]. However, under pathological conditions, APP is aberrantly processed through the amyloidogenic pathway, leading to the generation of neurotoxic fragments such as Aβ and APP-C31.

The burden of neurodegenerative diseases continues to grow with the ageing of global population profiles. According to Alzheimer’s disease International, approximately 10 million new cases of AD are reported globally each year [8]. The World Health Organization (WHO) also reported over 8.5 million cases of PD in 2019 [9]. Despite significant research efforts, current therapeutic strategies remain insufficient in halting or reversing disease progression. This underscores the need for a deeper understanding of the molecular mechanisms involved in neurodegeneration.

While Aβ has been extensively reviewed, the APP-C31 fragment represents an underexplored but promising contributor to neurotoxicity and disease progression. This review aims to synthesize current knowledge about APP-C31, including its generation, mechanisms of toxicity, regulation, and relevance to neurodegenerative conditions. This review would also provide a hypothesis of the central position of APP-C31 in the pathogenic cascade of neurodegenerative disease which indicates that APP-C31 should be considered as a critical mediator. A better understanding of APP-C31 may provide new avenues for therapeutic intervention.

## C31 fragment of APP (APP-C31)

### Structure of APP-C31

**Fig. 1:**
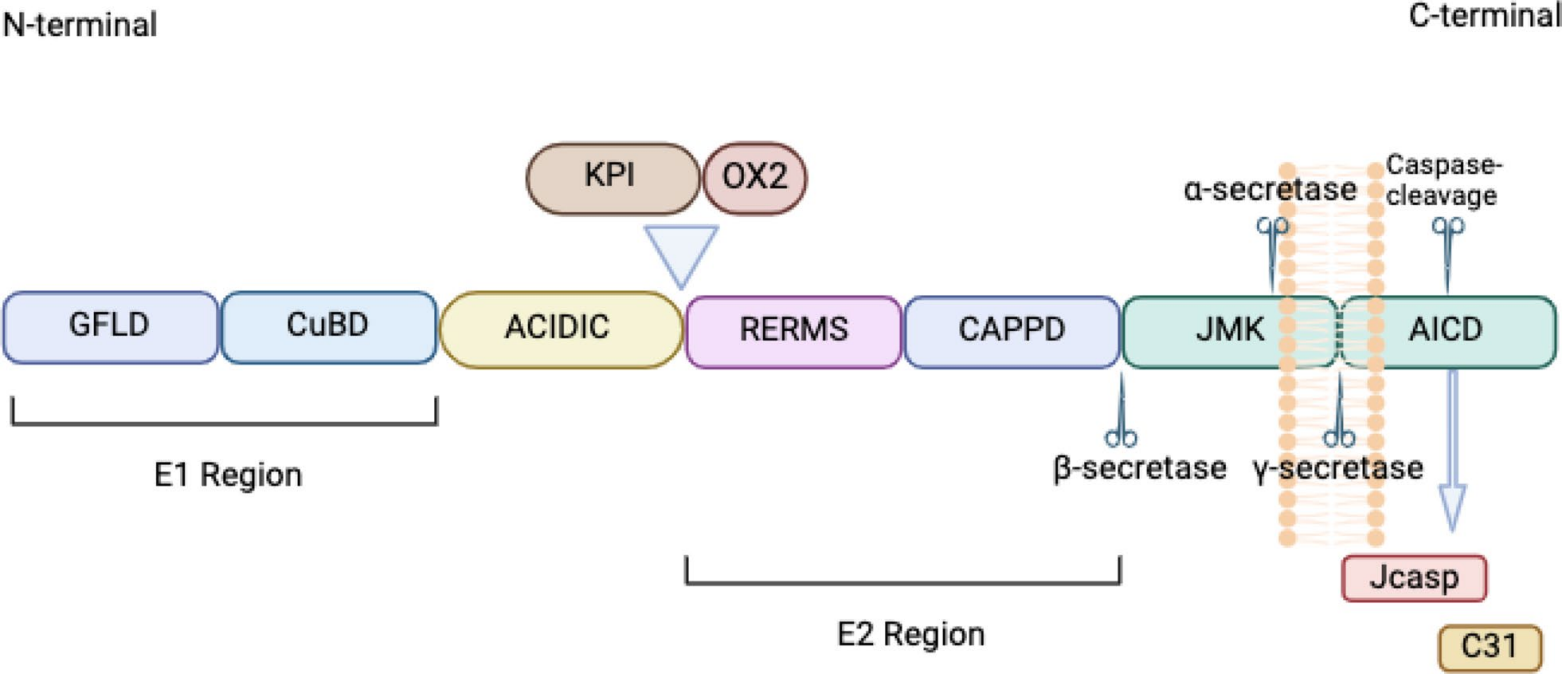
General structure of APP. The E1 region can be divided into two domain components, the growth factor-like domain (GFLD) and the copper-binding domain (CuBD). The E2 region consists of the arginine-glutamate-arginine-methionine-serine (RERMS) sequence and the central APP domain (CAPPD). An acidic domain (ACIDIC) exists between regions E1 and E2. The juxtamembrane kinase domain (JMK) and the APP intracellular domain (AICD) are both located in the region near the C-terminus of the APP. AICD can be a regulator of gene expression, cell apoptosis and cellular calcium homeostasis in the nucleus [14]. The Kunitz-type protease inhibitor (KPI) and the thymus-derived lymphoid OX-2 antigen homologue (OX2) may not exist in every isoform of APP. Among the three common isoforms of APP, only APP 770 contains both the KPI and OX2. APP 751 contains only KPI, whereas APP695 does not contain both KPI and OX2. Among the three common isoforms of APP, APP695 is expressed predominantly in neurons [13]. This figure was created with https://BioRender.com

APP consists of numerous regions, including two dimerization domains (E1 region and E2 region), an acidic amino acid-enriched (ACIDIC) region, a juxtamembrane domain, a transmembrane domain and an amyloid precursor protein intracellular domain (AICD) [10–12]. Moreover, APP has 11 isoforms, with APP695, APP751 and APP770 being three of the most common forms of APP [12, 13]. There are several differences between these three isoforms (Fig. 1).

**Fig. 2:**
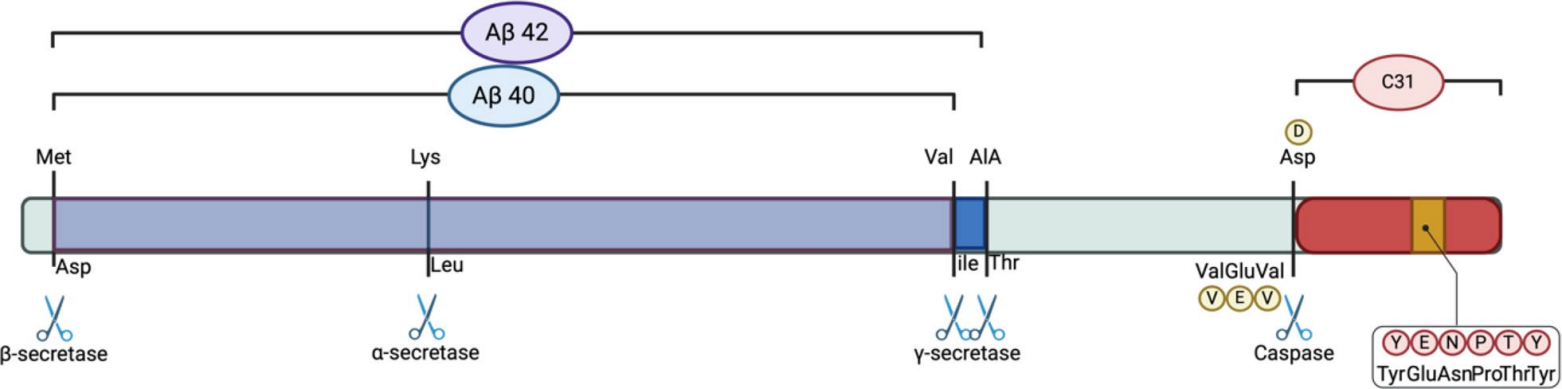
Structure of CTFβ (C99) and the cleavage sites for the production of the Aβ and C31 fragments. This figure was created with https://BioRender.com

In APP intracellular trafficking, full-length APP is commonly proteolysed into various fragments by different proteolytic enzymes and secretases. There are 3 cleavage sites on APP that can be cleaved by the α, β and γ secretases. Cleavage can be divided into trophic peptide formation, or the nonamyloidogenic pathway, and antitrophic peptide formation, or the amyloidogenic pathway [11]. The cleavage of APP by the α secretase results in the formation of sAPPα and an 83 amino acid α-C-terminal fragment (αCTF or C83), which can be further cleaved by γ secretase to generate P3. sAPPα and P3 are considered trophic peptides [11]. Therefore, cleavage by the α secretase is usually considered the trophic peptide formation or nonamyloidogenic pathway, as no amyloid is formed from this cleavage. On the other hand, cleavage by β secretase results in the formation of sAPPβ, which is considered an antitrophic peptide [11], and a 99 amino acid β-C-terminal fragment (βCTF or C99). The C99 fragment is further internalised and cleaved by γ secretase to form Aβ and AICD [12, 15] (Fig. 2). Moreover, AICD is also formed when γ secretase cleaves C83 to generate P3 [15]. AICD can be further cleaved by caspases-3, -6, -8 and -9 to finally form the Jcasp and C31 fragments [13]. These fragments are considered anti-trophic peptides that may lead to neurodegeneration [16]. Therefore, cleavage by the β secretase is considered an antitrophic peptide formation or amyloidogenic pathway.

### C99 fragment

C99, which is also known as C100, is the fragment generated by the cleavage of APP with β-secretase [17]. It has been shown to be further cleaved by caspases and γ-secretase and ultimately generate Aβ and C31 fragments [17]. As mentioned, the toxicity of Aβ depends not only on the presence of C31 but also on APP. Aβ forms a complex with APP, which can ultimately recruit and activate caspase cleavage and release C31 fragments. Similarly, Aβ can also form complexes with C99 fragments and facilitate the formation of C99 homomeric complexes [18]. The oligomerisation of C99 also recruits caspases to generate C31 fragments [18]. Therefore, the formation of C31 would be enhanced in the presence of the Aβ-C99 complex. In addition, C99 can also be cleaved to release C31 fragments, and this cleavage more easily occurs in thinner membranes [19]. Moreover, C99 may also induce the deposition of Aβ [20]. However, with the addition of α-secretase inhibitors, β-secretase inhibitors and pancaspase inhibitors, the amount of C99 increases, whereas the amounts of C31 and C83 decrease [21]. This may be due to other types of proteolytic processing [21]. Moreover, the amount of C99 and AICD decreased with the addition of brefeldin A [22]. The interaction between C99 and C83 may have an inhibitory effect on the production of Aβ [23], which may indirectly decrease the release of C31 fragments. In addition, the neurotoxicity of C31 is dependent on C99 fragments, which form a heterodimer of the C1‒C99 complex [24]. Although C99 does not directly generate C31, the Aβ produced by it serves as a crucial upstream driver for C31 generation.

### Aβ and APP-C31

Aβ and C31 fragments are believed to be neurotoxic and highly related to neurodegeneration in the brain, as Aβ can cause cellular perturbations, including neuron and glial death, increase the level of oxidative stress and decrease energy availability [25] in cultured cells. Moreover, the neurotoxicity of C31 and Aβ depends on their interaction with APP [26]. Additionally, the interaction between Aβ and APP promotes APP aggregation [18]. In fact, there are 2 forms of Aβ that can be produced during the cleavage of APP: Aβ40 (with 40 amino acids) and Aβ42 (with 42 amino acids) [15]. Moreover, at low concentrations, Aβ may not exert neurotoxic effects in the brain [27]. Indeed, Aβ monomers may be neuroprotective [28], while Aβ40 can promote the proliferation of neural progenitor cells and increase the survival of developing neurons via self-renewal [29]. However, when Aβ aggregates or is highly concentrated, Aβ40 may induce an inflammatory response by promoting the expression of inflammatory genes [30], and Aβ42 can directly induce apoptosis by activating the p53 promoter [31]. Moreover, Aβ42 may be more likely to promote amyloid plaque generation [32, 33]. Therefore, the balance between Aβ40 and Aβ42 is a critical factor in the pathogenesis of Alzheimer’s disease. Moreover, research has shown that the C31 fragment may selectively enhance the recovery and production of Aβ42 without altering total Aβ recovery, which may cause nonapoptotic cellular insult [34]. As a result, C31 may cause a change in the ratio of Aβ40 to Aβ42. Some researchers have speculated that the underlying mechanism may be a negative regulatory mechanism [34]. X11 family proteins may bind to βAPP to decrease the production of Aβ, but C31 may compete with X11 and bind with βAPP, which can further promote the recovery of Aβ42 [34]. However, this speculation may need further investigation.

Moreover, C31 can also enhance the neurotoxicity of Aβ40 in the brain. C31 may promote the neurodegeneration induced by Aβ40 by causing the reduction and fragmentation of neurites [35, 36]. C31 may also intensify the inflammatory response induced by Aβ40 [35]. In addition, C31 has been shown to facilitate and accelerate the aggregation of Aβ40 in the brain, especially in the nuclear and perinuclear regions [35, 36]. This may be because in the presence of C31, Aβ40 unfolds the α-helical segment, which may facilitate the β-sheet formation, a structural transition essential for the aggregation of Aβ [35–37]. Moreover, Aβ40 binds with C31, which can promote the generation of amyloid fibrils [36]. Therefore, the neurotoxicity of Aβ decreases when C31 is removed [38]. In contrast, the effect of the C31 fragment on Aβ42 may not be significant, as the polymerisation of Aβ42 is much faster [36]. Moreover, the toxicity of both C31 and Aβ requires the -GYENPTY- amino acid motif, which is a sequence within C31 peptides [18, 39]. Some researchers have proposed that this is because AICD or C31 may form a complex with Fe65 and CP2/LSF/LBP1 to induce the expression of GSK-3β, which is related to apoptosis [40], with the recognition site of Fe65 binding to form the complex being the NPTY domain in the -GYENPTY- motif [18]. Therefore, neurotoxicity relies on the -GYENPTY- motif. However, as the proposed idea is based on observations of C58 and C59, which are not physiologic AICDs, further proof is needed [18]. In addition, the NPTY domain also serves as an internalisation motif, which is essential for the production of Aβ [41].

### Caspase cleavage of APP

**Fig. 3:**
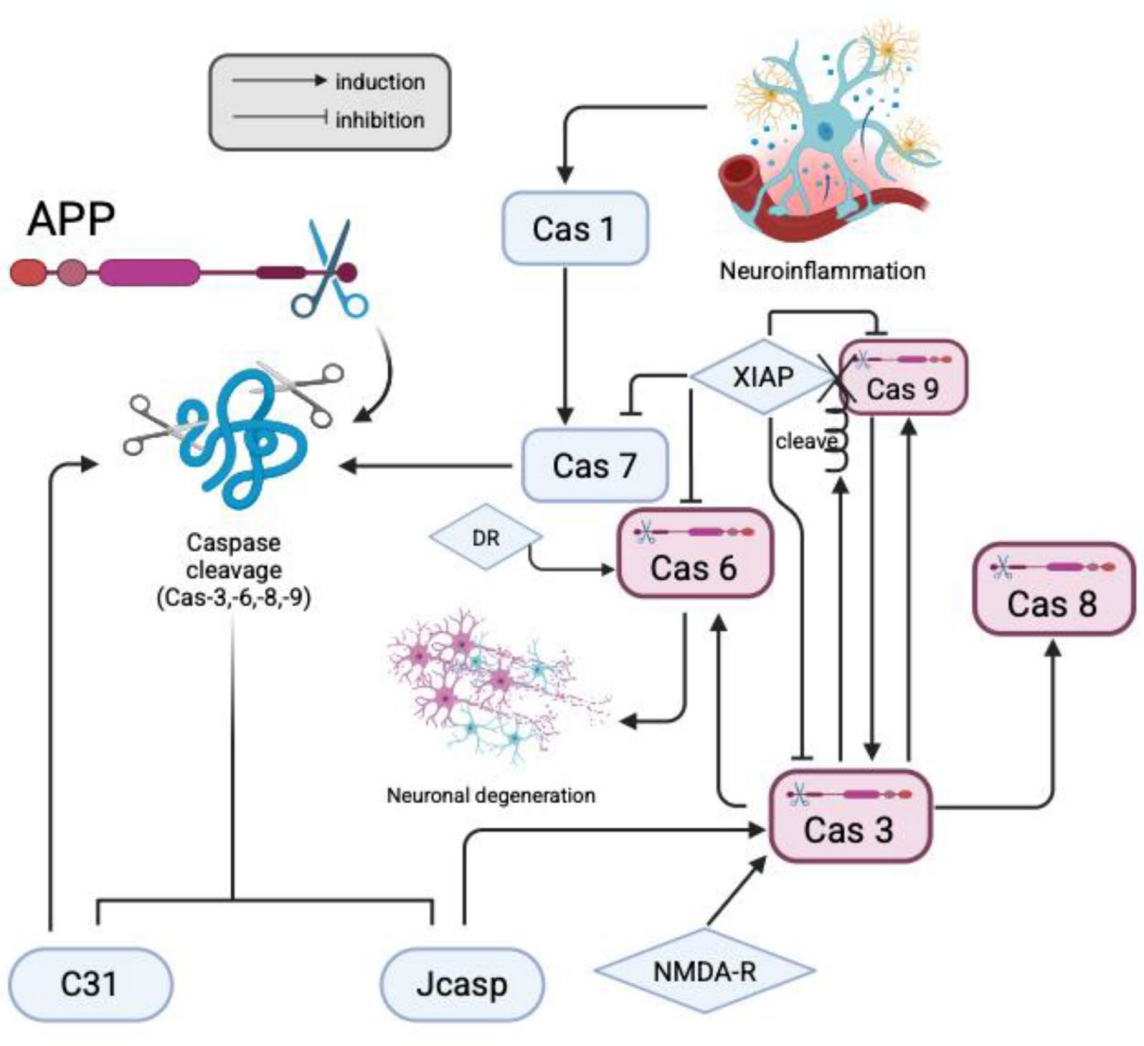
A graphical summary of the caspase’s interaction and the correlation with C31 fragments generation. This figure was created with https://BioRender.com

Caspases are known as a family of cysteine proteases in many animals [42]. Generally, caspases can be categorized into 2 groups on the basis of their functions: initiator and effector caspases [42]. The initiator caspases can affect the effector caspases and other substrates after dimerisation [42]. Studies have shown that caspase activation may contribute to cell death, but more evidence is needed to determine whether caspase activation initiates the death of neurons [43]. APP is believed to be the substrate of caspases, and it can be cleaved by caspases 3, 6, 8, and 9 (see Fig. 3). In the generation of C31 fragments [44], APP is usually cleaved at the Asp664 cleavage site by caspase-8 and caspase-9 [7]. Moreover, caspase-3 may be the effector and substrate of caspase-8 [45], whereas caspase-9 may also activate caspase-3. Moreover, caspase-3 cleaves caspase-9, which removes the X-linked inhibitor of apoptosis protein (XIAP) from caspase-9 [46]. XIAP may inhibit the activities of caspase-3, caspase-6, caspase-7 and caspase-9 [47]. Therefore, caspase-3 may also indirectly promote the activation of caspase-9. Furthermore, C31 may also inhibit XIAP, which can also indirectly promote the activation of caspase-9 [48]. In addition, studies have shown that in addition to full-length APP, αCTF and βCTF can also be substrates of caspases [7]. However, it is also reported that an inhibitor of caspase-9 does not inhibit neuronal apoptosis, whereas inhibitors of caspase-3, -6 and -8 can inhibit apoptosis [49]. In addition, studies have shown that APP is a substrate of caspase-3 [50], and some believe that the cleavage of APP is mainly by caspase-3 at the VEVD cleavage site [51–56]. Caspase-3 may also be involved in inflammatory signalling [57]. Moreover, C31 may also amplify the activation of caspase cleavage, especially that of caspase-8 [7, 38]. Additionally, caspase-3 can also activate caspase-6, which can cleave APP to generate C31 fragments by cleaving caspase-6 at the DVVD, TETD and TEVD sites [58, 59], while caspase-6 may be inhibited by zinc [60]. Caspase-6 is also the only caspase that is activated by DR6, leading to neuronal degeneration [61]. On the other hand, the caspase cleavage of APP generates not only C31 but also Jcasp, while Jcasp is also neurotoxic [13]. Jcasp may activate caspase-3, which can also cleave APP to release C31 fragments and Jcasp [62]. This self-amplifying mechanism promotes the accumulation of C31 fragments and Jcasp [62]. In addition to Jcasp, NMDA-R activation may also activate caspase-3, which is involved in the signalling cascade involved in the induction of long-term depression (LTD) [51]. Moreover, serum-deprived conditions and the overexpression of APP may also cause the activation of caspases and the generation of C31 fragments [63]. On the other hand, the overexpression of Bcl-2, which is a protein that can prevent the release of apoptotic factors from the mitochondria, may prevent the caspase cleavage of APP [64].

In addition, caspase-7 may also cleave APP to generate C-terminal fragments of 25 kDa, which can be activated by caspase-1, which is known to be associated with neuroinflammation [65]. Therefore, caspase-7 may be considered neuroinflammation-dependent and may also promote the cleavage of APP to generate C31 [66] and inhibit other neuroprotective pathways [65]. The following table summarizes the importance and role of each caspase (Table 1).

**Table 1:** Summary of the distinct role and importance of each caspase

Caspase	Role and importance
Caspase-1	Activated by neuroinflammation and further activate caspase-7
Caspase-3	Cleave APP at VEVD cleavage site. Effector and substrate of caspase-8 and activate caspase-6 directly. Also activate caspase-9 indirectly
Caspase-6	Cleave APP at DVVD, TETD and TEVD sites. The only caspase can induce neuronal degeneration
Caspase-7	Promote the caspase cleavage of APP to generate APP-C31 fragments and inhibit other neuroprotective pathways when activated
Caspase-8	Cleave APP at Asp664 cleavage site
Caspase-9	Cleave APP at Asp664 cleavage site. Activate caspase-3

### Aβ, caspase induction and C31

**Fig. 4:**
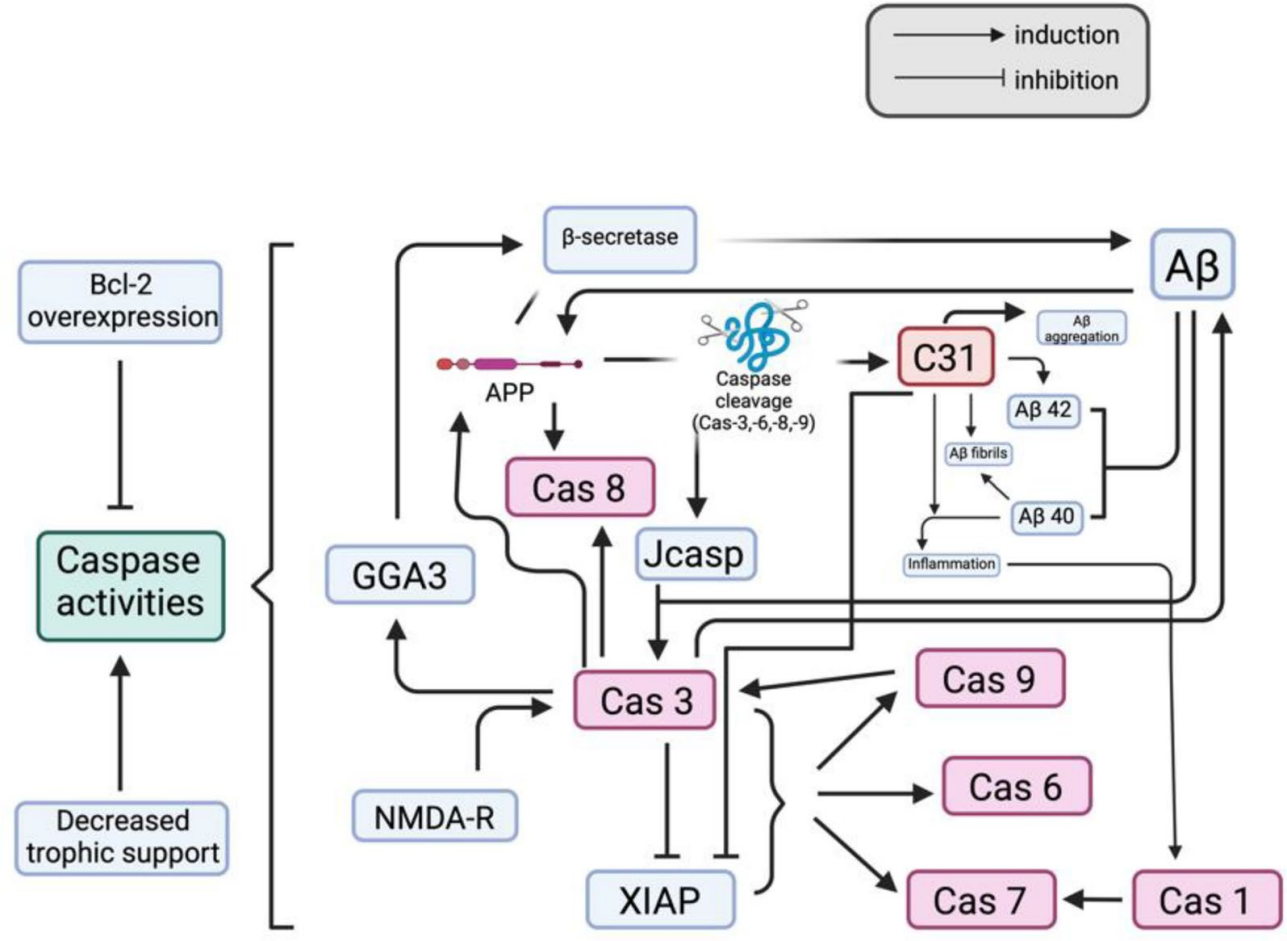
A graphical summary of the relationships among Aβ, C31 fragments and caspases. This figure was created with https://BioRender.com

The toxicity of both Aβ and C31 is believed to be mediated by caspases 8 and 9, while the cleavage of these caspases to generate C31 fragments may be activated by soluble Aβ [67, 68]. Therefore, the initiation of the caspase cleavage of APP may be responsible for the neurotoxicity of Aβ [67]. In addition, the cleavage of APP may be the intermediate step of the neurotoxicity of Aβ [26, 68]. In other words, the toxicity of Aβ is attenuated in the absence of the C31 fragment [7, 69]. C31 fragments participate in a positive pathological feedback loop with Aβ, secretases, and caspases, as described in detail in Sect. 3.5. Notably, C31 also appears to act as an amplifier of caspase activation [7]. Consequently, under pro-apoptotic stressors, including exposure to Aβ, the presence of C31 increases the likelihood of neuronal cell death [7]. In contrast, in the absence of C31, Aβ can still exert intrinsic toxicity, but without C31-mediated signal amplification and the reinforcing pathological loop, its harmful effects are attenuated. Consistent with this model, studies have shown that expression of a caspase-resistant form of APP reduces Aβ toxicity [67]. Aβ may form complexes with APP and accelerate the multimerisation of APP, which can further recruit and activate caspase 8 to release C31 fragments [60, 68]. Moreover, the process of complex formation may also be amplified by nonfibrillar Aβ [68], which also explains the belief that soluble Aβ can activate the caspase cleavage of APP to generate C31. Some believe that caspase cleavage may not have contributed to the increased amount of Aβ [70], as it is unlikely to be involved in the amyloidogenesis pathway of APP, whereas the generation of Aβ may be more dependent on the β-site APP cleaving enzyme 1 (BACE1 or β secretase) [71]. Therefore, even if the cleavage site is removed, the generation of Aβ is not affected [69, 71]. In contrast, some researchers have reported that the overexpression of APPΔC31, a product produced after the C31 peptide sequence is removed from full-length APP, may decrease the production of Aβ [41]. This may be because APPΔC31 lacks an internal signal, which is included in the C31 fragment, and because the processing of APP [44] and amyloidogenesis require the endocytosis of APP. These conflicting findings may be partially explained by a pathway in which caspase cleavage can indirectly affect the generation of Aβ [44]. As active caspase 3 cleaves Golgi-localized γ-ear-containing ARF binding protein 3 (GGA3), which can traffic BACE for lysosomal degradation, caspase 3 prevents BACE degradation and thus increases Aβ production [44, 72]. However, APPΔC31 may also exert neurotoxicity by inducing caspase-3-independent apoptosis [63]. Moreover, caspase-3 can also increase the generation of Aβ by directly interacting with APP [65]. Moreover, Aβ-dependent synaptotoxicity may also cause the activation of caspase-3 in synapses. Caspase-3 further activates calcineurin, which can dephosphorylate the GluR1 subunit of AMPAR to remove AMPAR. The removal of AMPAR may cause an increase in LTD [73]. The summarized relationship among Aβ, caspase induction and C31 is illustrated in Fig. 4.

## Regulation of the generation of C31 fragments

### Trophic support

As mentioned, the cleavage of APP ultimately leads to the generation of C31 fragments, which can induce neuron death. Cellular death is commonly regulated by signals received by the receptor of the cells. The absence of the trophic ligand or the presence of the antitrophic ligand would probably induce cellular death via the generation of neurotoxic components, including C31, Jcasp and Aβ. One example of a trophic factor of APP is netrin-1 [11, 74]. Netrin-1 can bind with APP and Aβ, which can ultimately lead to a reduction in Aβ generation [74]. Moreover, with sufficient trophic support to neurons, APP is cleaved mainly through the trophic peptide formation (nonamyloidogenic) pathway [11]. Therefore, adequate trophic support is important for cell survival and preventing the aggregation of neurotoxic components. In contrast, when trophic support is reduced and not enough to support neuron survival, the ratio of the trophic peptide formation (nonamyloidogenic) pathway to the anti-trophic peptide formation (amyloidogenic) pathway shifts, and APP is mainly cleaved by β-secretase, which leads to an increase in the generation of C31 [11]. Moreover, Aβ may also be an anti-trophic ligand that can reduce trophic support to neurons [75].

### ASK1

Studies have shown that the ASK1 and JIP1 adaptor proteins and the JNK protein are present in the immunoprecipitated complex with APP [76], which indicates that there is some relationship between ASK1 and APP. ASK1 is an apical MAPKKK in the mitogen-activated protein (MAP) kinase signalling cascade, while JIP1b binds with the C31 fragment to induce neuronal death [77]. ASK1 is critical for the induction of stress-induced neuronal cell death, and ASK1 can also upregulate the activity of an inhibitor of the proteasome (E2-25K/Hip-2), which is implicated in Aβ-induced neurotoxicity [78]. Moreover, the induction of Aβ toxicity may also be dependent on JNK1 and p38, which are effectors of ASK1 [77]. When trophic support is reduced or oxidative stress is increased, ASK1 is upregulated by the activation of MAPKKs [77]. Moreover, ASK1 is implicated in the neuronal death induced by the dimerization of APP and EGFR (epidermal growth factor receptor) [79, 80]. Therefore, ASK1 may constitute the underlying cascade involved in the neuronal response to the reduction in trophic support to generate C31 fragments.

### Adaptor protein 4

Adaptor protein 4 (AP-4) is a member of the heterotetrametric adaptor protein family and is a complex with four subunits in total [81]. Studies have shown that the subunit (μ4) of AP-4 interacts with APP at the YKFFE sequence site [79]. The interaction between AP-4 and APP may also affect the amount of cleavage products of APP. When the interaction is disrupted, the cleavage of APP by γ-secretase increases, which further decreases the amount of CTFα and CTFβ and increases the levels of Aβ and AICD. Moreover, C31 generation decreases [79]. In addition, depletion of the AP-4 subunit μ4 may also cause an increase in Aβ secretion [79].

### G protein

G proteins are a group of proteins that are critical in signal transduction and second messenger cascades [82]. The G protein binds with APP at the site of the 657–674 region of APP, and this binding is regulated by C31 fragments [83]. The -YENPTY- domain within the C31 fragment is essential for the release of the Go subunit [83, 84]. Moreover, the cleavage of APP to generate C31 also activates the G protein signalling pathway [83]. G proteins are also activated when Aβ binds to APP, and the G protein is released [83]. The G protein signalling pathway results in an increase in the calcium level in the cell, which can ultimately cause the death of the cells [83]. The activation of the G protein by APP also regulates sodium currents and increases the phosphorylation of APP (Thr668) through the activation of JNK [85]. Moreover, the G protein and p21 activated kinase (PAK3) also mediate the neuronal apoptosis caused by C31 fragments [86], while PAK3 interacts with C31 fragments to induce apoptosis [87]. APP can also interact with the heterotrimeric G protein Gαo [88]. The hyperstimulation of the interaction between APP and Gαo can induce the chronic elevation of reactive oxygen species (ROS) [89]. The elevation of ROS can subsequently activate the pro-apoptotic caspases, caspase 3, 7 and 9 [89] which can further cleave APP to generate C31 fragments. In addition, G-protein coupled receptors can also activate the PI3K/Akt pathway through the Gα or Gβγ subunit [90]. The activation would further phosphorylate caspase 9 which may also contribute to the generation of C31 fragments [91]. Studies also discovered that the overexpression of G-protein couple receptor 3 would promote Aβ generation independently without affecting the expression of γ-secretase [92].

### Positive feedback pathologic loop

Taken together, a positive feedback loop for generating C31 fragments has been illustrated. When trophic support to neurons is reduced, APP is more readily cleaved by β-secretase and γ-secretase, which is followed by caspase cleavage to finally release C31 fragments. Moreover, Aβ and C99 fragments are also produced. The Aβ-C99 complex promotes the production of C31, and C31 also promotes the generation of Aβ fibrils, which can increase β-secretase levels [93, 94]. In addition, the activation of caspase-3 during the production of C31 indirectly promotes the amount of β-secretase. With increasing levels of β-secretase, more APP can be cleaved to further generate Aβ, C99 and C31 fragments.

## Mechanism of APP-C31 cytotoxic induction of apoptosis

**Fig. 5:**
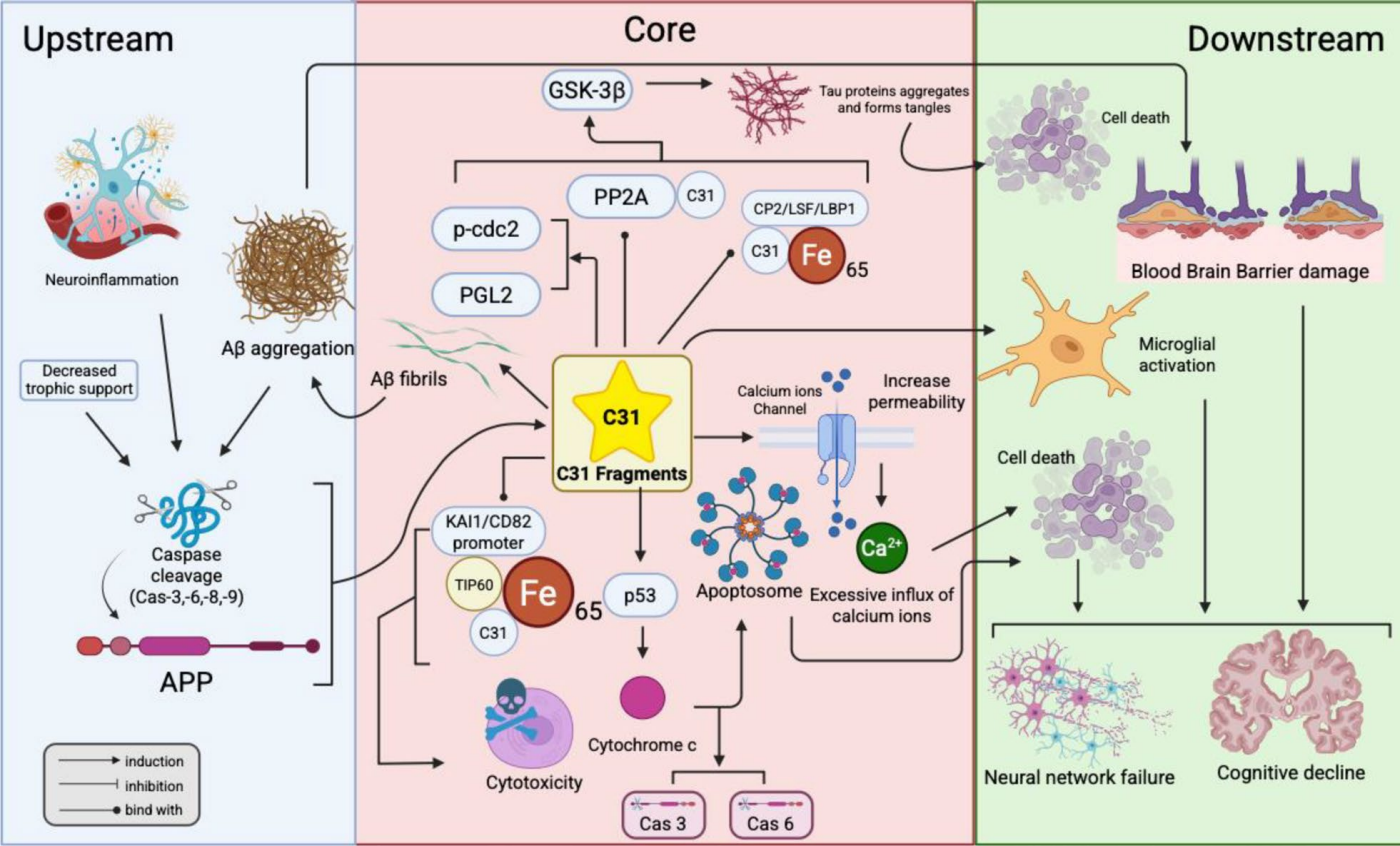
A graphical summary of the position of C31 fragments in the pathogenic cascade and cell death induction pathway indicating that C31 fragments should be considered as a critical facilitator. This figure was created with https://BioRender.com

It is well known that the overexpression of C31 fragments leads to cell death [95]. There are several ways by which C31 fragments induce neurotoxicity, which ultimately leads to neuronal apoptosis and neurodegeneration. Fig. 5 illustrates the critical position of C31 in the pathological cascade.

### GSK-3β pathway

GSK-3β is a kinase that plays a role in the metabolism of glycogen, and it is also expressed in the central nervous system. The major pathway in which GSK-3β is involved is the phosphorylation of various proteins, including tau protein, mitochondrial pyruvate dehydrogenase and c-Jun [96]. In the context of the overexpression of GSK-3β, the tau protein undergoes hyperphosphorylation, and neurons undergo apoptosis [97]. C31 translocates into the nucleus and induces the transcription of GSK-3β [98]. Studies have shown that the release of C31 can induce the expression of GSK-3β by increasing promoter (PGL2) activity and p-cdc2 activity, which is the promoter of cdc2 [40]. In addition, C31 also binds with PP2A at the NPTY region in the -YENPTY- domain, whereas PP2A is a phosphatase that mediates the phosphorylation of tau proteins [99]. C31 may also induce the expression of GSK-3β and subsequent tau hyperphosphorylation by forming a complex with Fe65 [100], an adaptor protein, and CP2/LSF/LBP1 in the nucleus [40]. The hyperphosphorylation of the tau protein impairs its function, which can no longer stabilize the microtubules [101]. Moreover, the tau protein also aggregates and forms tangles [101]. Neurons with tau tangles exhibit hyperactivation of calcium ion-dependent proteases and calcium ion-activated kinases, which results in abnormally high levels of calcium ions in the cells [102]. Therefore, tau protein tangles ultimately lead to the death of neurons [101].

### Ternary complex formation

C31 can also form a complex with Fe65 and Tip60, which is a histone acetyltransferase [103]. C31 can bind Fe65 at the -YENPTY- domain [104]. The ternary complex binds with the KAI1/CD82 promoter, which competes with and displaces the N-CoR/TAB2/HDAC3 complex in the absence of the IL-1β signal [40]. This binding could further cause the activation of target genes to exert cytotoxicity [40].

### Ion homeostasis disruption

The accumulation of C31 fragments may alter the permeability of the calcium channel in neurons [105] by enhancing the current of the voltage-gated calcium channel [106]. The calcium channel becomes more permeable, which allows excessive influx of calcium ions into the neuron [105]. An excessive amount of calcium ions in cells ultimately leads to cell death [105]. C31 may also induce the release of cytochrome c from the mitochondria, which leads to the apoptosis of the cells [105]. Cytochrome c can further bind with Apaf-1, which results in the formation of apoptosomes [107]. In addition, C31 increases the production of nitric oxide in microglia and astrocytes [105].

### p53

Fragments released from the γ-secretase cleavage of APP may induce the expression of p53 [108]. Meanwhile, it is believed that p53 is an AICD-dependent regulated gene in inducing cell death via the AICD/FE65 transcriptional regulation [108]. Additionally, Tip-60 also regulates p53 [109]. ACID can also interact with p53 mRNA which can further promote the expression of p53 and p44, a shorter isoform, to activate the p53/p44-mediated proapoptotic signaling pathways [110]. p53 is a gene encoding a transcription factor that can transcriptionally regulate genes involved in the cell cycle and apoptosis [111]. The level of p53 increases when DNA is damaged, and cells are under oxidative stress [111]. An increased level of p53 causes the activation of proapoptotic genes, such as Bax [111]. Bax binds with the bcl-2 family and interacts with the mitochondrial voltage-dependent anion channel [111]. This interaction further causes leakage of the mitochondrial membrane and leads to the release of cytochrome c [112]. Meanwhile, the activation of Bax by p53 can also further activate a death-inducing platform, apoptosome which would finally cause the activation of caspase-3, through the activation of caspase-9 [113]. Moreover, p53 can also directly promotes the transcription of caspase-6 [114]. Caspases-3 and -6 would further generate C31 fragments to enhance the neurotoxicity.

## Diseases related to the APP-C31

### Alzheimer’s disease

**Fig. 6:**
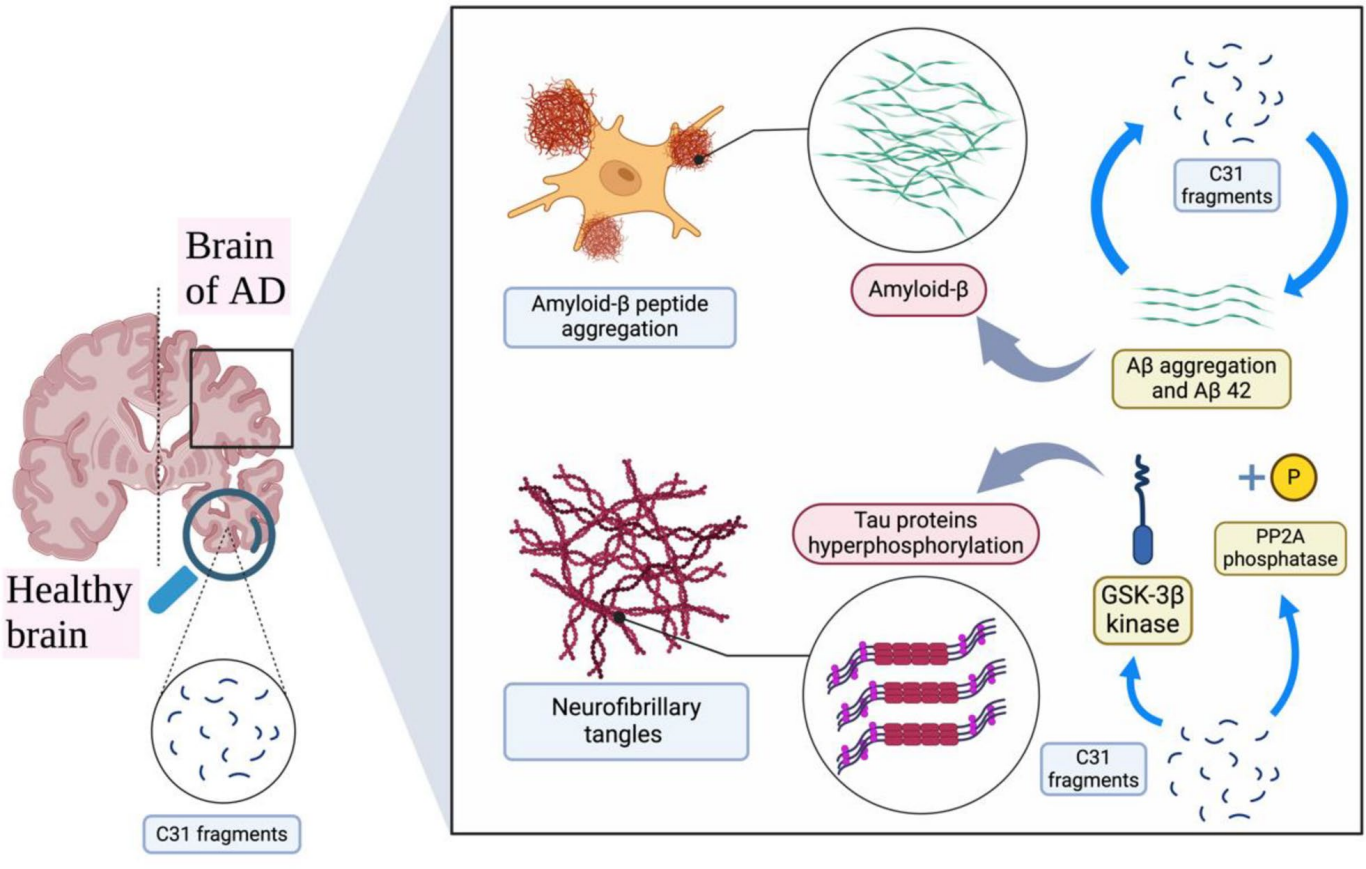
Graphical summary of the pathology of AD and its relationship with C31. This figure was created with https://BioRender.com

Alzheimer’s disease (AD) is characterized by the progressive loss of learning and memory, which ultimately leads to the loss of greater cognitive function [115]. Two hallmarks of AD are believed to exist: extracellular aggregations of the amyloid-β peptide (Aβ) and intracellular neurofibrillary tangles [116], which are formed by the hyperphosphorylation of tau proteins [117]. Studies have also shown that C31 fragments are present only in the brains of AD patients but not in those of controls [118]. Moreover, the underlying cellular mechanism of the formation of memory is related to synaptic plasticity [119]. Synaptic plasticity can occur in two main forms: long-term potentiation (LTP) and long-term depression (LTD) [119]. LTP is responsible for memory formation, whereas LTD is responsible for memory loss [120]. In addition, brain-derived neurotrophic factor (BDNF) is a neurotrophin in the CNS that is important for promoting LTP and inhibiting LTD [120]. Neurotrophins work with their receptors, including p75 neurotrophin receptors and tyrosine kinase receptors (TrkA, TrkB, and TrkC) [120]. In addition, Aβ may also have an inhibitory effect on LTP in the hippocampus [102]. Moreover, it is believed that NMDA-R calcium ion influx is associated with both LTP and LTD [121], whereas Aβ-induced LTP inhibition may rely on NMDA-Rs [122, 123]. However, Aβ may also affect LTD and LTP in the brain through other mechanisms [124]. As mentioned above, C31 fragments also increase the level of Aβ, which further increases the inhibitory effect of Aβ on LTP. On the other hand, AICD can also promote LTD and inhibit LTP, which depends on C31 fragments [51]. Therefore, APP, as the precursor protein of neurotoxic components (Aβ, AICD, and C31) involved in many other neuronal pathways, is highly relevant in the pathology of AD (Fig. 6).

#### Familial Alzheimer’s disease (FAD)

Most cases of late-onset AD are not related to genetic defects, whereas patients with familial AD (FAD), characterized by early-onset AD, commonly present with particular genetic defects or mutations [125]. Mutations can be found in the genes encoding APP or presenilin (PSEN1 and PSEN2) [125]. Presenilin is a protease of γ-secretase [125]. Two types of mutations in APP have been discovered: Swedish mutation and Flemish mutation [126]. In Swedish mutation, the sensitivity of β-secretase is increased, which could cause APP to be cleaved more frequently and lead to the excessive generation of Aβ and subsequent C31 fragments [127]. Moreover, the cleavage site of γ-secretase is also mutated, which may also cause increased generation of Aβ and C31 [127]. With Flemish mutation, the sensitivity of α-secretase decreases, which makes APP more difficult to cleave by α-secretase to generate a trophic peptide [126]. Therefore, APP may be cleaved by β-secretase more easily. On the other hand, the overexpression of mutated PSEN1 and PSEN2 may increase susceptibility to apoptotic stimuli, including Aβ exposure [128]. In addition, mutations in PSEN1 or PSEN2 may also increase GSK-3 activity, hyperphosphorylation of the tau protein and activation of caspase-3 through interference with phosphatidylinositol-3-kinase (PI3K)/Akt signalling activation [129]. Nevertheless, PSEN1 mutation may also disrupt calcium ion homeostasis in neurons by increasing the generation of Aβ and reducing the production of sAPPα [102]. An increased level of Aβ impairs calcium influx via voltage-dependent channels and Aβ-forming channels via the induction of oxidative stress [102].

#### Importance of C31 fragments in AD

The importance of C31 fragments in the pathogenesis of AD has been increasingly recognized. In transgenic mouse models engineered to remove the caspase cleavage site on APP, thereby preventing the generation of C31 fragments without altering Aβ production, researchers observed a striking phenotype [130]. Despite the presence of Aβ deposits, these mice did not exhibit AD-like features, including dentate gyrus (DG) atrophy, reduced hippocampal presynaptic densities, astrogliosis, enhanced neuronal progenitor proliferation, spatial and working memory deficits, or neophobia [130]. These findings suggest that C31 fragments, rather than Aβ deposition alone, may play a more critical role in driving key pathological and behavioural manifestations of AD.

#### The multifaceted role of APP-C31 in the AD pathogenic cascade

C31 functions not as the initial trigger of AD, but rather as a critical amplifier of disease progression and phenotypic converter, acting predominantly downstream of or in parallel to Aβ pathology to drive the crucial transition from amyloid accumulation to neuroinflammation, severe synaptic dysfunction, and neuronal death. In this hierarchical framework, C31 operates through distinct, context-dependent roles. Primarily, it serves as a central mediator within intracellular signalling pathways, where it acts as a direct effector of toxicity via caspase-3 and -8 activation, nuclear translocation altering gene expression, and the induction of mitochondrial dysfunction, as discussed in the previous sections. Simultaneously, it functions as a facilitator and synergistic factor in intercellular communication, amplifying the damage caused by Aβ and tau pathology by exacerbating microglial activation, through the stimulation of Stat1 inflammatory pathway [131], and compromising the blood–brain barrier, through the amplification of Aβ which can subsequently activate Death Receptor 6 disrupting the Wnt/β-catenin and JNK pathways [132, 133]. While C31 may initially appear as a secondary bystander, arising merely as one of several by-products of γ-secretase cleavage during the intense Aβ generation of early-stage disease, its subsequent stability and potent intrinsic toxicity rapidly elevate it beyond a passive marker, establishing it as a key driver of the disease's catastrophic later stages.

### Parkinson’s disease

**Fig. 7:**
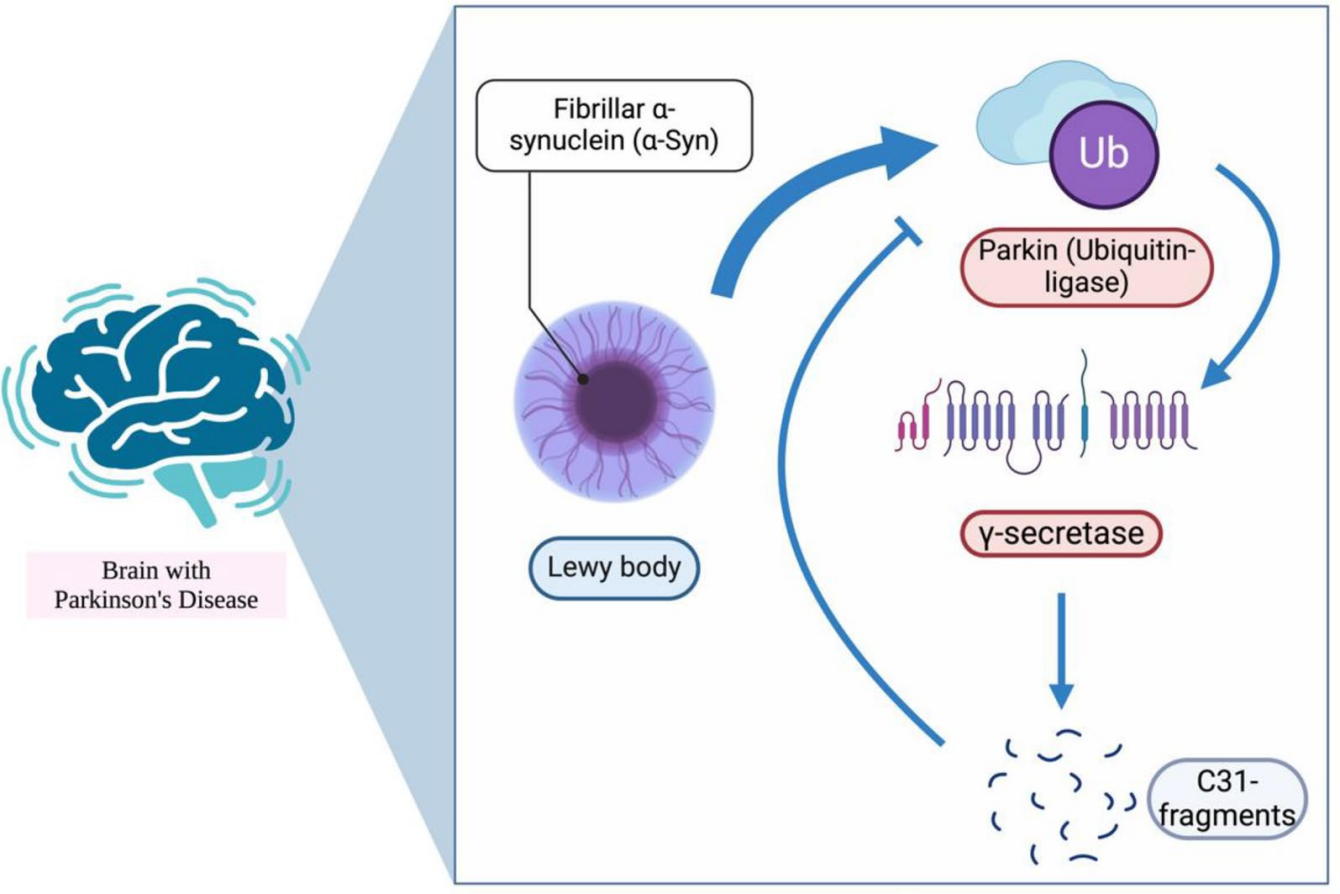
Graphical summary of the relationship between C31 and PD. This figure was created with https://BioRender.com

Parkinson’s disease (PD) is another neurodegenerative disease caused by the degeneration of dopaminergic neurons in the brain [134]. The hallmark of PD is believed to be the aggregation of fibrillar α-synuclein (α-Syn), which is also known as a Lewy body [134, 135]. The aggregation of α-Syn is similar to Aβ aggregation, but the hydrophobic domain of α-Syn may also induce α-Syn to form amyloid filaments [134]. This aggregation triggers the ubiquitin‒proteasomal system, which is associated with the ubiquitin‒ligase parkin [136, 137]. Some studies have demonstrated that parkin can be a transcription factor [138] that can increase the level of γ-secretase [139] and thus increase the production of Aβ and C31 fragments (Fig. 7). However, some studies have shown that the intracellular accumulation of Tau proteins, which may be caused by C31 fragments, may decrease the level of parkin [140], whereas parkin-associated phenotypes may exert a protective effect on Aβ toxicity by increasing Tau degradation [141].

### Other related diseases

#### Traumatic brain injury (TBI)

**Fig. 8:**
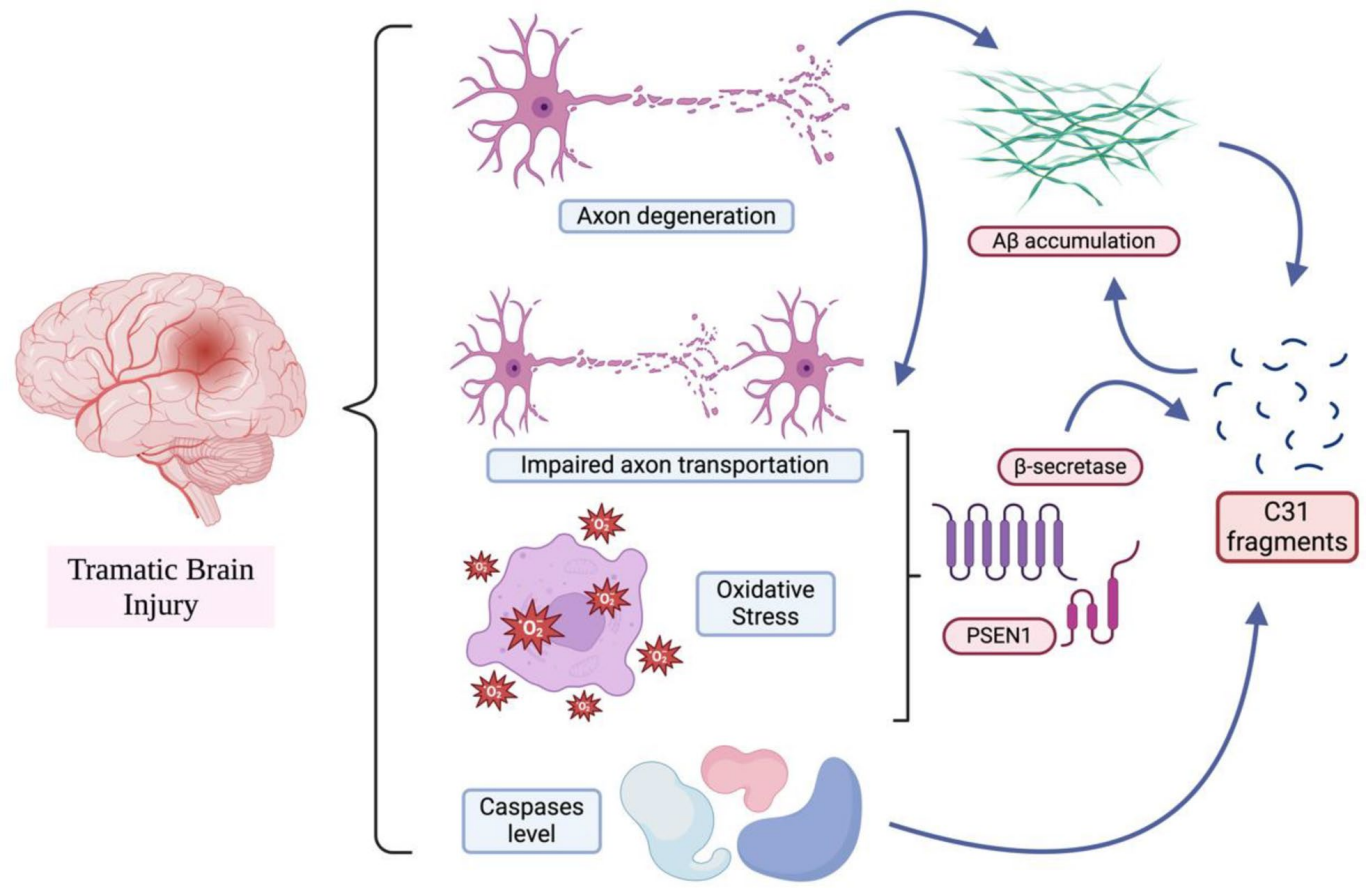
Graphical summary of the relationship between TBI and C31. This figure was created with https://BioRender.com

Traumatic brain injury (TBI) refers to brain disability in terms of cognitive ability or physical function after a certain degree of damage [142]. It is believed that TBI is one of the risk factors for AD, as studies have shown that approximately 30% of TBI patients accumulate Aβ, β-secretase and PSEN1 [143]. The increase in and accumulation of Aβ may be due to the degeneration of axons [144], whereas the increase in β-secretase and PSEN1 may be due to impaired axon transportation or oxidative stress caused by injury [145]. Moreover, TBI may also cause elevated levels of caspase-1, -3 and -8 [146, 147], and caspase may be activated by the influx of calcium ions into neurons [148]. As mentioned, caspase-1 may activate caspase-7, which can promote the generation of C31 fragments, whereas caspase-3 and caspase-8 can cleave APP directly to generate C31 and Aβ afterwards (Fig. 8). Therefore, these factors may cause secondary damage to the brain, which may increase the risk of the onset of AD.

#### HSV-1 infection

**Fig. 9:**
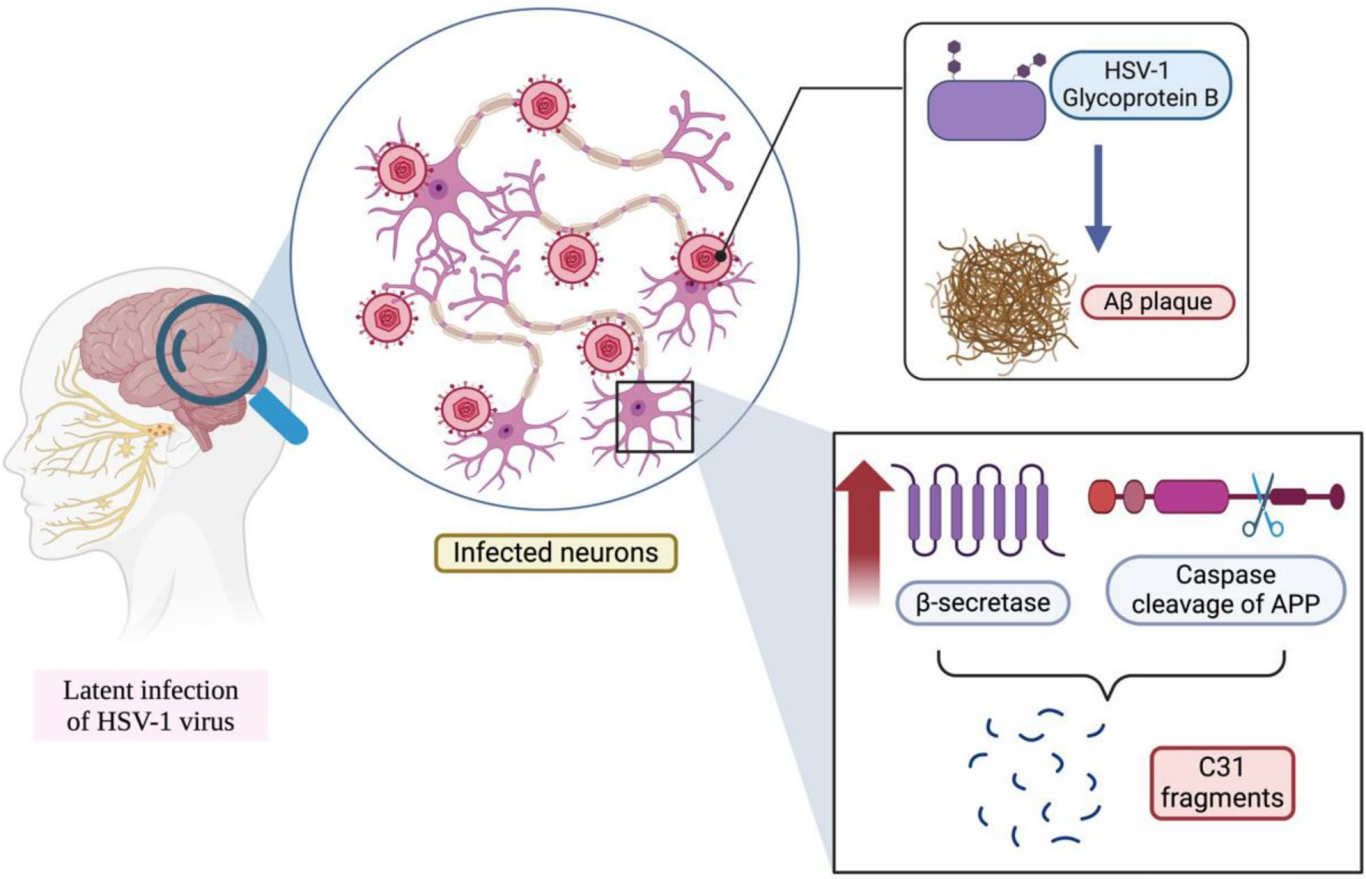
Graphical summary of the pathology of HSV-1 infection and its relationship with C31. This figure was created with https://BioRender.com

Herpes simplex virus type 1 (HSV-1) is a neurotropic double-strand DNA virus [149]. Infection with HSV-1 usually causes lesions on the epithelium of the oral or nasal mucosa [149]. Moreover, HSV can also cause latent infection, which can infect the nervous system through reactivation [150]. One of the viral proteins, HSV-1 glycoprotein B, is similar to Aβ and thus can initiate the accumulation of Aβ to ultimately form Aβ plaques [151]. Moreover, HSV-1-infected neurons express elevated levels of β-secretase, and HSV-1 may also induce the cleavage of APP, which promotes the generation of C31 fragments [150] (Fig. 9). Moreover, the HSV-1 virus may also be able to alter calcium ion channels to increase intracellular calcium ion signalling [149].

#### Down syndrome (DS)

**Fig. 10:**
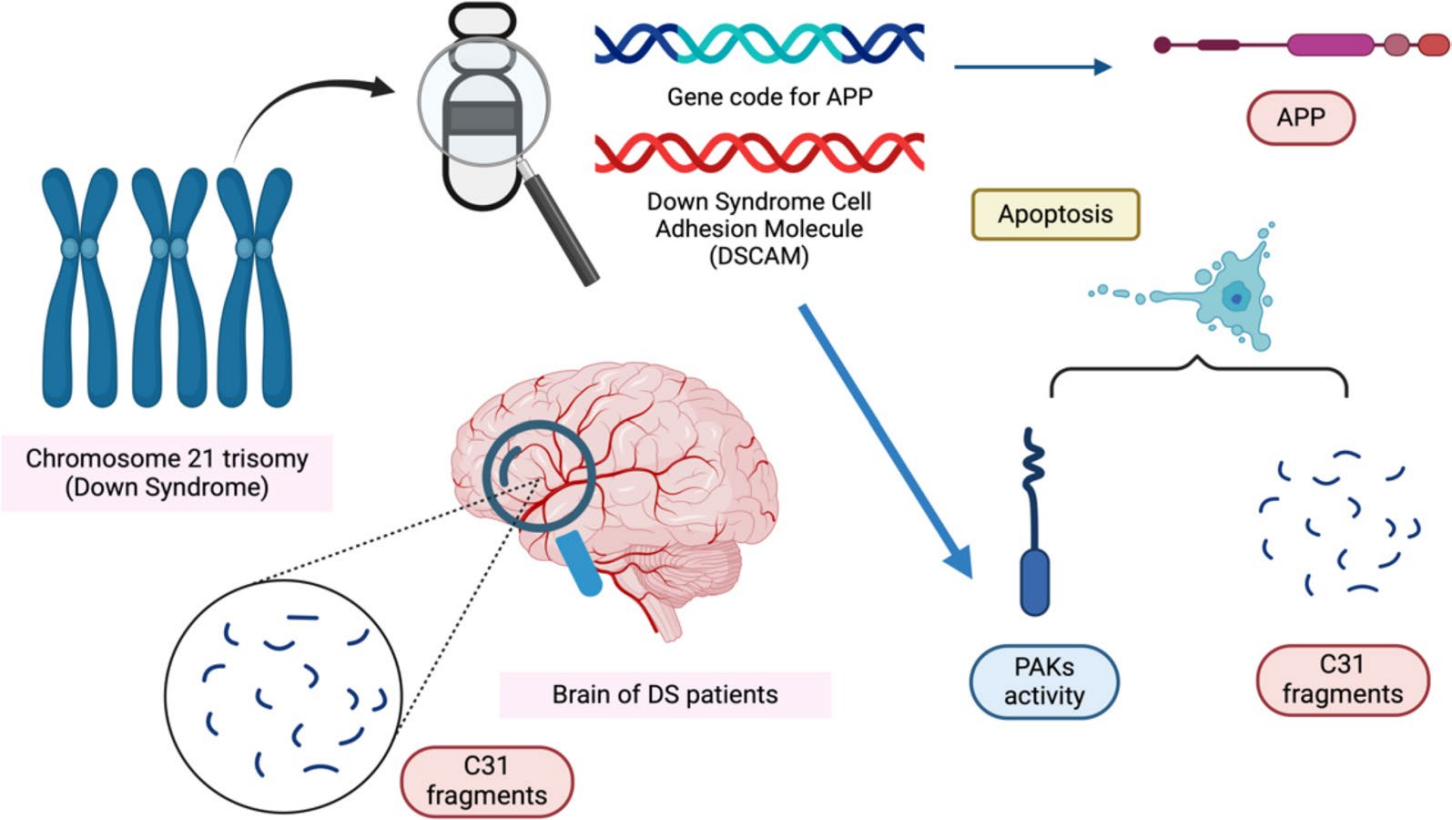
Graphical summary of the relationship between C31 and DS. This figure was created with https://BioRender.com

Down syndrome is a genetic disease caused by the trisomy of chromosome 21. Moreover, the genes encoding APP are also located on chromosome 21, whereas DS patients usually exhibit early-onset AD. This may be due to the overexpression of APP coding genes, which are trisomy chromosomes, in DS patients [152]. Moreover, the cleavage of APP and the number of C31 fragments are also increased in the brains of DS patients [153]. One of the DS-related genes is Down Syndrome Cell Adhesion Molecule (DSCAM) [153]. When DSCAM is overexpressed, the generation of neurons, synaptogenesis, axonal outgrowth and neurite arborization are strongly affected [153]. In addition, DSCAM is involved in the regulation of PAK activity, while PAKs are activated by DSCAM [153] (Fig. 10). Thus, the overexpression of DSCAM would cause hyperactivation of PAK activity, while PAKs are one of the downstream effects of APP processing that cause neuronal death [152].

## Potential treatments for AD related to C31 fragments

### APP phosphorylation at Thr668

APP can be phosphorylated by Cyclin-dependent kinase 5 (Cdk5) [154], cdc2 kinase and c-jun N-terminal kinase (JNK) in the brain [155]. Phosphorylation is important for the normal physiological function of the brain and neurons, including neuron outgrowth. This phosphorylation causes a conformational change in APP at the Thr668 site, altering the bond between sites 668 and 669 [156]. This conformational change may lead to alterations in the interaction of APP with other proteins. Studies have shown that phosphorylated APP leads to a reduction in APP cleavage by caspase-3 and caspase-8 [157]. As mentioned above, both caspases can cleave APP directly and release C31 fragments from APP. In addition, phosphorylated APP may also affect the formation of a ternary complex with Fe65, as the conformation of APP is altered [158]. Therefore, phosphorylated APP may also contribute to reducing the toxicity of APP by decreasing the production of C31 fragments and the formation of a ternary complex.

### Antagonists of G protein activity

As previously mentioned, the activation of the G protein may play a critical role in regulating the death of neurons and neurodegeneration. The G protein is believed to trigger cell death by mediating membrane permeability to calcium ions, which results in excessive influx of calcium ions into cells. Moreover, G protein activation may also promote excessive release of calcium ions from the endoplasmic reticulum (ER) [159], which further increases the level of calcium ions in the cell and ultimately causes cell death. Nevertheless, this mechanism may also serve as a positive feedback loop, as the increased influx of calcium ions may promote the generation of Aβ [160–162]. An increase in Aβ production causes an increase in C31 generation, while C31 can also activate the G-protein signalling pathway. An increase in Aβ enhances the interaction between Aβ and APP, which can cause the activation of G proteins. As a result, the influx of calcium ions is further amplified by the C31 fragments and Aβ. Therefore, antagonists of the G protein may inhibit the effects caused by the activation of the G protein and the initiation of the G protein-mediated positive feedback loop. Thus, the neurodegeneration caused by C31 and Aβ through the G protein-activating pathway may be reduced.

### NMDA-R blockade

The NMDA receptor (NMDA-R) may also play an important role in the pathology of AD. Research has shown that the activity of the NMDA-R may affect the level of APP cleavage by α-secretase, which may promote the cleavage of APP by α-secretase and the generation of C83 or αCTF [163], which are trophic peptides. Moreover, some studies have demonstrated that NMDA-R activity may also decrease the production of Aβ40 [163]. However, studies have demonstrated that NMDA-R activity can activate caspase-3 and ultimately cause LTD [51]. As the activation of caspase-3 cleaves APP to generate C31 fragments and triggers a series of effects that can further amplify the toxicity to neurons, LTD of the brain can occur. In addition, some studies have reported that prolonged activation of NMDA causes shifting of the APP isoform from APP695 to KPI-containing isoforms, such as APP770 and APP751, while Aβ generation is also elevated [13, 164]. Therefore, NMDA-R blockade may be helpful in attenuating neurodegeneration in AD by inhibiting the activation of caspase-3 and increasing the production of Aβ via NMDA-R activity, which would help reduce the generation of C31 fragments. However, α-secretase cleavage may also be inhibited or reduced.

### β-secretase inhibitors

In the previous sections, the role of β-secretase in the pathology of AD and the generation of C31 fragments and Aβ was illustrated, and the cleavage of APP by β-secretase can be considered the initiating point of the amyloidogenic pathway. Therefore, researchers and companies are working on inhibitors of β-secretase to inhibit the amyloidogenic pathway of APP processing. Some β-secretase inhibitors, including thalidomide and nifedipine, have already been tested in AD patients [165, 166]. In addition, β-secretase cleavage site inhibition by site-directed monoclonal antibodies may also be effective at inhibiting β-secretase cleavage [167]. Therefore, the inhibition of β-secretase reduces the levels of Aβ and C31, which can directly reduce neurodegeneration events. Although these inhibitors may seem to be a more mature strategy for preventing AD, several difficulties need to be addressed, including the specificity and toxicity of the drugs [168].

### Cell cycle inhibition and disruption of APP endocytosis

When the cell cycle is activated, the levels of PHF-1, APP and cyclin B are elevated, while increased cyclin B can bind with cdc2 to induce the phosphorylation of tau proteins [111]. As mentioned, C31 fragments also induce the phosphorylation of tau proteins, so cell cycle activation may contribute to the hyperphosphorylation of tau proteins and increase C31 toxicity. Moreover, cell cycle activation may also trigger the phosphorylation of APP and amyloidogenesis, as phosphorylation may increase the number of endocytic vesicles and is related to amyloidogenic processing [111]. Therefore, the amyloidogenesis triggered by cell cycle inhibition increases the generation of C31 fragments and causes neurodegeneration. Studies have shown that cell cycle progression inhibitors, which block the G1/S phase transition, may reduce the phosphorylation of APP [169]. However, when the inhibitors block the M-phase, the phosphorylation of APP and the amyloidogenic processing of APP increase [169].

### mChiAICD

The chimeric AICD mutant (mChiAICD) is designed to be able to eliminate the cleavage site of γ-secretase and caspase-3 [120]. mChiAICD can produce a protein with a similar-YENPTY domain [120] and thus can interact with γ-secretase and caspase-3, which compete with APP to bind with γ-secretase and caspase-3. Therefore, it would be able to prevent APP cleavage by γ-secretase and caspase-3, which would generate C31 fragments and decrease C31 generation. Moreover, mChiAICD may also reduce the activity of GSK-3β, which can further decrease tau protein phosphorylation [120]. This may help compensate for the induction of GSK-3β activity by C31 fragments to reduce the neurotoxicity of C31 fragments. Nevertheless, mChiAICD can also reduce the level of Aβ42 in neurons by facilitating the clearance of Aβ42 and the inhibition of γ-secretase [120]. The reduced level of Aβ42 may also help reduce the generation of C31 fragments, as Aβ42 may also increase the generation of C31 fragments. mChiAICD can also increase the levels of BDNF and antiapoptotic Bcl-2 proteins [120].

### sAPPα and αCTF

sAPPα and αCTF are considered trophic peptides that can stimulate cell growth and differentiation and are crucial for cell survival. sAPPα is also considered a neuroprotective and antiapoptotic fragment [65]. Research has shown that mutation of the CuBD domain of APP may increase the sAPPα level, which can provide cells with trophic support to reduce the cleavage of APP by β-secretase [10]. Additionally, a decreased level of Aβ is also observed in mutated APP [10]. In addition, reports have shown that sAPPα may also be a possible endogenous inhibitor of β-secretase, which can effectively reduce the toxicity of APP. Moreover, research has shown that sAPPα can effectively increase LTP induction [170]. Clinically, studies also have discovered several factors that may affect the production of sAPPα. Estrogen is one of the examples which can alter the metabolism of APP to shift towards the nonamyloidogenic pathway [171] by stimulating the activity of α-secretase selectively [172]. In nonamyloidogenic pathway, the generation of sAPPα is promoted as mentioned in the previous section. Therefore, the generation of C31 fragment, via the amyloidogenic pathway, would be decreased. As a result, the neurotoxicity induced by C31 fragments can also be reduced while the neuroprotective effect from sAPPα would be increased. Therefore, it can be concluded that the neuroprotective properties of sAPPα offer two key clinical translation perspectives in AD, firstly, as a biomarker of pathophysiological equilibrium, secondly, by enhancing its levels through modulating α-secretase selectively, this represents a therapeutic paradigm of 'empowering the brain's own protective mechanisms, complementing strategies that directly counteract toxic proteins.

### Caspase cleavage inhibitors

As mentioned, caspase cleavage plays an important role in the positive pathological feedback loop and the generation of C31 fragments. Therefore, the inhibition of caspases may help alleviate the neurodegeneration of neurons by directly inhibiting the cleavage of APP to generate C31 fragments. The overexpression of Bcl-2 may be an inhibitory strategy that can block the activation of caspases [64]. In addition, spiperone and thapsigargin can inhibit the activation of the Wnt signalling pathway, which is important in cell apoptosis [173]. However, inhibition of the Wnt pathway may be effective only in the early stage of AD [173]. Moreover, a SERCA inhibitor can significantly reduce C31 production in response to treatment with statins [173], and calcium channel blockers may also be effective in preventing the influx of calcium ions [173].

### Grb2-SH2 interaction

Growth factor receptor binding protein-2 (Grb2) contains a Src homology 2 (SH2) domain that can interact with the -YENPTY- domain on AICD directly [174, 175]. Moreover, studies have shown that the interaction between Grb2-SH2 and APP can rapidly transport AICD, which can prevent the accumulation of AICD in neurons and the cleavage of AICD by caspases [176]. Therefore, the interaction may reduce the generation of C31 fragments in the neurons.

### Posiphen tartrate

Posiphen tartrate is an inhibitor of the synthesis of APP [177]. As APP has been shown to be the precursor protein of toxic components, including C31 fragments and Aβ, which can induce the pathological pathway of AD, inhibiting the synthesis of APP to reduce the level of APP may be helpful for reducing the release and generation of subsequent neurotoxic components. Moreover, the first phase trial of Posiphen tartrate revealed the ability of Posiphen tartrate to reduce the Aβ, sAPPβ and tau protein levels in the central spinal fluid [178]. However, the level of trophic sAPPα is also reduced [178]. Further investigations are needed to determine whether it is effective in AD patients [179].

### Chaperone-mediated autophagy (CMA)

Chaperone-mediated autophagy is a selective form of autophagy that can transport targeted components to lysosomes to degrade these components [180]. Moreover, APP contains the motif targeted by CMA, so CMA should be able to eliminate APP in cells [180]. Therefore, CMA would be able to eliminate the APP and prevent its cleavage, which would generate C31 fragments. However, the degradation of the calcineurin 1 regulator (RCAN1) by CMA may increase susceptibility to the pathogenesis of AD [181].

### ASBI-1 (rutin) and ASBI-2 (galangin)

ASBI refers to AβPP-selective β-secretase inhibitors, which include ASBI-1, rutin, ASBI-2, and galangin [182]. Instead of directly inhibiting β-secretase, ASBI binds with AβPP to prevent the cleavage of AβPP by β-secretase [182]. Therefore, the inhibitory effect is AβPP-selective, which can reduce the production of the subsequent release of the neurotoxic Aβ and C31. Additionally, ASBI-2 may also be an inhibitor of acetylcholine esterase, which can induce autophagy [183, 184]. Thus, similar to CMA, ASBI-2 may also help eliminate APP in cells to prevent the cleavage of APP and the generation of C31 fragments.

### Antioxidants

Carnosic acid, obtained from a neutral product, is an antioxidant that may contribute to protection from oxidative stress [185]. Carnosic acid may reduce the level of Aβ and the oligomerization of Aβ [186]. Moreover, Aβ increases the activation of caspase-3, which further increases the generation of C31 fragments through the cleavage of APP by caspases. Therefore, carnosic acid may reduce the level of C31 fragments through the reduction of Aβ. However, the actual effect of carnosic acid on the C31 fragment may need further clarification [186]. On the other hand, another antioxidant, β-estradiol, may also have a neuroprotective effect, which can inhibit the C31-induced current enhancement of voltage-gated calcium channels and reduce the toxicity of C31 fragments [106].

**Fig. 11:**
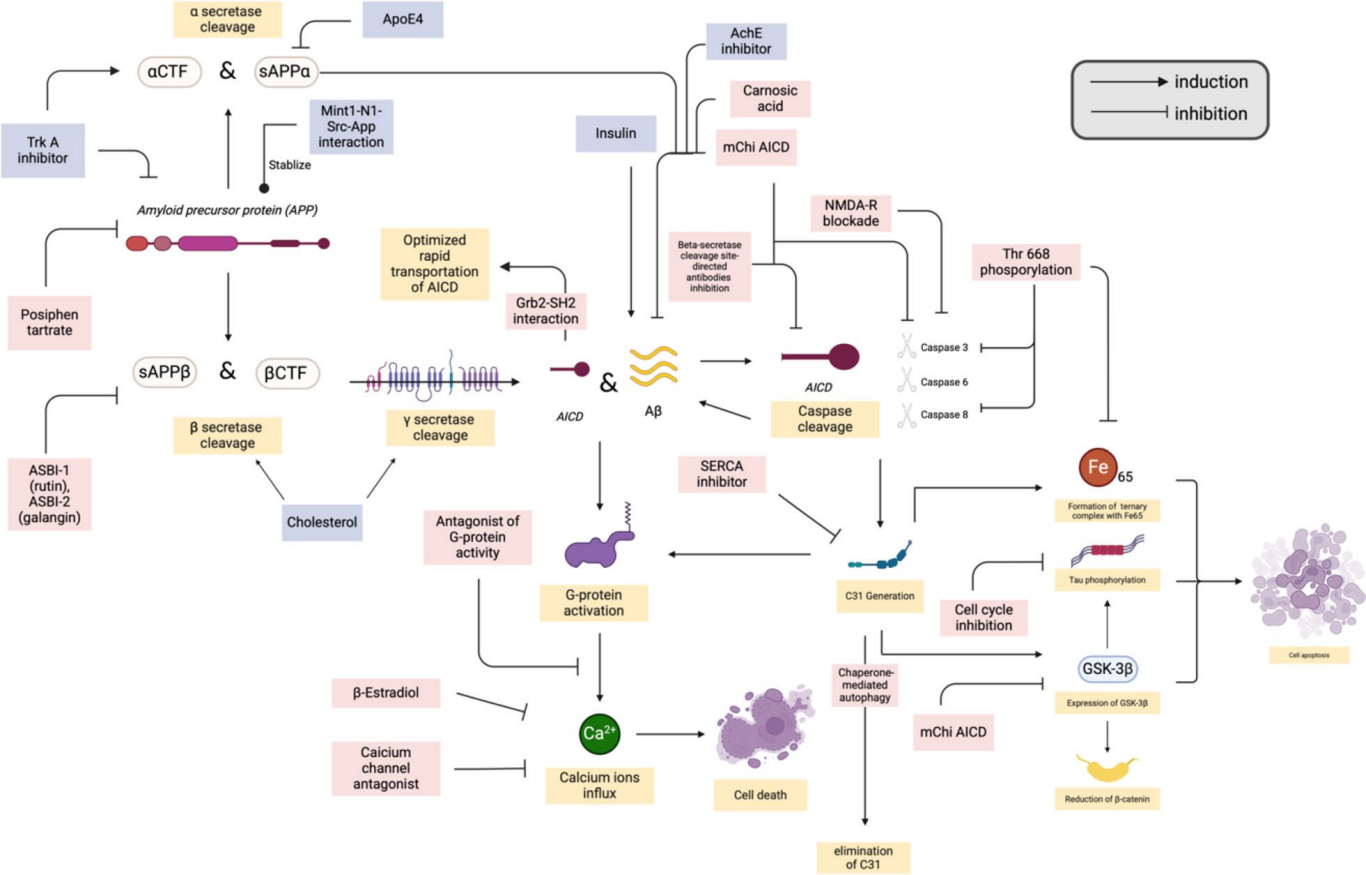
Summary of the potential treatments for AD and other factors affecting the generation of C31 fragments. This figure was created with https://BioRender.com

The overall relationship between C31 fragments and potential treatments of AD is shown in Fig. 11.

### Clinical application of C31 fragments

The accumulation and deposition of Aβ may not correlate with neuronal loss or the formation of neurofibrillary tangles, although it may constitute a risk factor for Alzheimer’s disease (AD) [43]. Moreover, the mere presence of Aβ deposits may not be sufficient to produce AD phenotypes [130]. Consequently, C31 fragments may represent more specific diagnostic and prognostic markers for neurodegenerative progression and severity. At present, however, the direct detection of C31 fragments is challenging to implement in clinical practice. In research settings, C31 fragments in neurons can be detected using antibodies specific to the N-terminal end of the C31 fragment [63]. Alternatively, some studies detect APP only after its cleavage using end-specific polyclonal antibodies [67]. These may potentially be applied to detect C31 fragments in post-mortem tissue clinically [11]. However, the direct detection of C31 fragments in CSF and serum may still be challenging and require further research.

Beyond their potential as biomarkers, C31 fragments may be more valuable as therapeutic targets, by inhibiting or disrupting the generation of C31 fragments. As discussed in Sect. 4, C31 fragments not only contribute to tau tangle formation but also participate in a positive pathological feedback loop with Aβ. Thus, therapeutically targeting C31 could simultaneously modulate both Aβ and tau tangle formation and disrupt this pathological loop. Meanwhile, targeting C31 fragments generation affecting the downstream of the pathological cascade which may offer a direct influence on the neuron degeneration. For example, administration of a pan-caspase inhibitor, which can prevent the generation of C31 fragments, has been reported to enhance long-term potentiation (LTP), a key mechanism underlying memory formation [187]. Therefore, C31-targeted therapies may prove effective by reducing Aβ and tau tangle formation, inhibiting the generation of symptom-contributing fragments, and enhancing memory-related synaptic plasticity. However, there are still some challenges in the clinical translation including the physiological role of its generation pathway, blood–brain barrier permeability, and optimal intervention timing.

Furthermore, several studies suggest that Aβ plaque burden may not directly account for neurodegenerative symptoms [43, 130], whereas C31 may play a more critical role. Therefore, APP-C31-directed strategies are not intended to clear Aβ plaques or tau tangles, but rather to block the downstream vicious cycle triggered by these initial pathologies, which leads to direct neuronal death, offering a distinct approach from existing anti-amyloid or anti-tau strategies. It may be particularly suitable for AD patients exhibiting distinct amyloid pathology and emerging neurodegenerative biomarkers or early cognitive decline. It may be considered as a part of combination therapy, used with the anti-amyloid or anti-tau therapy, in order to exert the greatest therapeutic efficacy.

## Other factors affecting the generation of C31

### TrkA overexpression

As in the previous paragraph, TrkA is one of the receptors of the neurotrophins. Studies have revealed that the overexpression of TrkA increases the levels of C31 fragments and Aβ [188]. In addition, Trk overexpression may cause the accumulation of NGF‒TrkA complexes, which may induce the phosphorylation of tau proteins to exert pathogenetic effects [188]. However, it should also be noted that the TrkA signalling pathway is multifactorial, as TrkA simultaneously activates the cleavage of APP and inhibits its cleavage [188]. Nevertheless, the overall effect of the overexpression of Trk is believed to be neurotoxic. Moreover, studies have shown that the inhibitor TrkA can lead to an increased ratio of sAPPα to Aβ, which has a beneficial effect [188]. Furthermore, treatment with a TrkA inhibitor may also reduce the level of APPneo [189].

### Insulin level

Insulin and IGF-1 signalling are important in mediating oxidative stress, neurotrophic factors, the phosphorylation of tau proteins and Aβ regulation [134]. Moreover, insulin resistance may impair the insulin/IGF-1 signalling pathway, which may cause pathological expression of GSK-3β [134]. On the other hand, insulin may also directly affect the accumulation of Aβ in the brain by promoting the generation of Aβ [134]. Moreover, insulin-degrading enzyme (IDE), which can degrade both insulin and Aβ, is inhibited by excessive levels of insulin [190]. Therefore, the level of Aβ increases, which can also cause increased generation of C31 fragments [190]. Furthermore, IDE may also cleave C31 fragments, so the inhibition of IDE may also directly increase the number of C31 fragments [191].

### Cholesterol level

It is believed that APP processing occurs in caveolae, which are membrane rafts off the cell membrane, while caveolae consist of cholesterol [134]. Therefore, when the level of cholesterol is excessively high, the number and size of caveolae, the expression of APP, and the activation of both β- and γ-secretase increase [134]. Therefore, the cleavage of APP is also increased, increasing the levels of Aβ and C31 fragments [134]. Moreover, the lipoprotein responsible for the transportation of cholesterol in caveolae, Apo E4, may also cause a decreased level of trophic sAPPα and increased formation of Aβ fibrils [192, 193]. Therefore, studies have revealed that statins, which are used to lower cholesterol levels, may also be effective at inhibiting β-secretase and promoting α-secretase [194, 195].

### Mint-N1-Src-App interaction

N1-Src and Mint1 expression may cause dimerization of APP, which stabilizes APP; thus, the level of full-length APP can be increased [196]. Moreover, as mentioned, the toxicity of C31 fragments also depends on the full-length APP, as C31 fragments may also interact with APP to exert toxicity. Therefore, the increased level of full-length APP may also contribute to the increased expression of C31 and the toxicity of C31.

### Anti-AchE inhibitors

Anti-AchE inhibitors, which are inhibitors of acetylcholinesterase (AchE), can facilitate cholinergic transmission and affect the generation, transmission and aggregation of Aβ [197]. Moreover, an inhibitor of butyrylcholinesterase (BuChE), an enzyme that hydrolyses acetylcholine in the synaptic cleft, may also promote cholinergic activity, restoring the AChE/BuChE activity ratio to exert therapeutic effects [197]. The decreased synthesis of Aβ would also help reduce the generation of C31 fragments.

## Others

### Hirano bodies

Hirano bodies are included in the cell and are F-actin rich [198], which may increase with increasing age [199]. Additionally, Hirano bodies are also observed in the brains of AD patients [199]. Some studies have suggested that Hirano bodies may promote the early processing of tau proteins, which may include the aggregation of tau proteins [200]. However, Hirano bodies are also believed to be neuroprotective in the brain, which may protect the brain from C31 fragment-induced tau protein hyperphosphorylation-related apoptosis as well as tau protein-independent apoptosis [198]. In addition, studies have shown that Hirano bodies can also protect cells from C31-induced cell death. Hirano bodies may decrease the level of GSK-3Aβ and affect the phosphorylation of tau proteins [201]. In addition, Hirano bodies can also colocalize with AICD and Fe65, which can further decrease transcription and apoptosis [202]. Nevertheless, Hirano bodies may also affect the cleavage of APP to generate C31 fragments [203].

### uMtCK and APLP1/C31 interaction

uMtCK is believed to be important in protecting mitochondria from toxins and oxidative stress [204]. Studies have revealed that when the expression of C31 fragments increases, the turnover of the uMtCk protein decreases, whereas the steady-state level of the uMtCk preprotein increases [205]. Therefore, uMtCK may exert beneficial effects on C31-overexpressing cells [204].

The APP-related gene product, APLP1, is cleaved by caspase-3, which potentially generates C31 fragments [206]. Additionally, the level of APLP1 may also increase in the presence of p53, which may be caused by oxidative stress [207]. Moreover, APLP1/C31 can also induce culture cell death [206] and increase the accumulation of Aβ extracellularly [207].

### Pyruvate dehydrogenase

Pyruvate dehydrogenase is a mitochondrial complex that can convert pyruvate to acetyl-CoA and NADH, which are important for the generation of ATP. In AD patients, the activity of PDH is decreased by 30%, possibly because the overexpression of β-secretase may reduce the level of PDH [208]. However, some studies have shown that the C31 fragments enhance PDH activity by binding with Nipsnap1 [208].

### APP-BP

APP binding protein 1 (APP-BP1) is the binding protein for APP, which acts as the regulatory subunit of the activating enzyme for the ubiquitin-like protein [209]. The expression of APP-BP1 may drive the cell division cycle through the S‒M checkpoint, while the overexpression of APP-BP1 leads to cell apoptosis [209]. Moreover, C31 fragments are believed to contain a motif that can bind and interact with APP-BP1 and further mediate neuronal cell cycle entry [210]. On the other hand, C31 fragments may also inhibit APP-BP2-mediated degradation by stabilizing substrates to reduce binding [211].

## Conclusions and future perspectives

In conclusion, it is believed that the APP-C31 fragments released from the cleavage of APP play an important role in the pathological pathway of neurodegeneration. Moreover, it can also be considered a therapeutic target in treatment by either reducing the generation of C31 fragments or reducing the toxic effect caused by C31 fragments through the inhibition of downstream signalling pathways.

However, some questions still need to be solved soon. Although the neurotoxic properties of C31 fragments have been discovered, the normal physiological role of C31 fragments is still unknown. Moreover, some underlying principles or pathways of the C31-related effect also need further study. More importantly, many therapeutic methods target C31 fragments to treat AD, but many of them are still at the theoretical level. Therefore, further studies are needed to determine the actual effects on AD patients.

## Data Availability

No datasets were generated or analysed during the current study.
